# Lifetime and point prevalence of psychotic symptoms in adults with bipolar disorders: a systematic review and meta-analysis

**DOI:** 10.1017/S003329172200201X

**Published:** 2022-10

**Authors:** S. R. Aminoff, I. N. Onyeka, M. Ødegaard, C. Simonsen, T. V. Lagerberg, O. A. Andreassen, K. L. Romm, I. Melle

**Affiliations:** 1 NORMENT, Division of Mental Health and Addiction, Oslo University Hospital & Institute of Clinical Medicine, University of Oslo, Oslo, Norway; 2 Early Intervention in Psychosis Advisory Unit for South East Norway, Division of Mental Health and Addiction, Oslo University Hospital, Oslo, Norway; 3 Department of Psychology, Sociology & Politics, Sheffield Hallam University, Sheffield, UK; 4 University of Oslo Library, University of Oslo, Oslo, Norway

**Keywords:** Adults, bipolar disorder, bipolar type I disorder, bipolar type II disorder, lifetime prevalence of psychotic symptoms, meta-analysis, point prevalence of psychotic symptoms, systematic review

## Abstract

Psychotic symptoms, that we defined as delusions or hallucinations, are common in bipolar disorders (BD). This systematic review and meta-analysis aims to synthesise the literature on both lifetime and point prevalence rates of psychotic symptoms across different BD subtypes, including both BD type I (BDI) and BD type II (BDII). We performed a systematic search of Medline, PsycINFO, Embase and Cochrane Library until 5 August 2021. Fifty-four studies (*N* = 23 461) of adults with BD met the predefined inclusion criteria for evaluating lifetime prevalence, and 24 studies (*N* = 6480) for evaluating point prevalence. Quality assessment and assessment of publication bias were performed. Prevalence rates were calculated using random effects meta-analysis, here expressed as percentages with a 95% confidence interval (CI). In studies of at least moderate quality, the pooled lifetime prevalence of psychotic symptoms in BDI was 63% (95% CI 57.5–68) and 22% (95% CI 14–33) in BDII. For BDI inpatients, the pooled lifetime prevalence was 71% (95% CI 61–79). There were no studies of community samples or inpatient BDII. The pooled point prevalence of psychotic symptoms in BDI was 54% (95 CI 41–67). The point prevalence was 57% (95% CI 47–66) in manic episodes and 13% (95% CI 7–23.5) in depressive episodes. There were not enough studies in BDII, BDI depression, mixed episodes and outpatient BDI. The pooled prevalence of psychotic symptoms in BDI may be higher than previously reported. More studies are needed for depressive and mixed episodes and community samples.

Prospero registration number: CRD 42017052706.

## Introduction

Bipolar disorders (BD) are severe mental disorders that are characterised by depressed and hypomanic/manic episodes, with varying degrees of euthymia in between mood episodes. The lifetime prevalence of BD is estimated to be 1–3% (Merikangas et al., 2011; Rowland & Marwaha, 2018). BD is subdivided based on the severity of the elevated mood into BD type I (BDI), which is characterised by manic episodes, and BD type II (BDII), which is characterised by hypomanic episodes. Psychotic symptoms, such as delusions and hallucinations, are common, particularly in manic episodes of BDI (Goodwin & Jamison, 2007). Although per their definition psychotic symptoms are incompatible with hypomania and euthymia, the prevalence of psychotic symptoms appears to vary between the other different types of mood episodes, that is, mania, mixed episodes and depression.

There are indications that psychotic BD is more severe than nonpsychotic BD, presenting with a poorer functional outcome (Bonnín et al., 2019), treatment adherence (Martinez-Aran et al., 2009) and lithium response (Hui et al., 2019). There are also indications of larger cognitive impairments in psychotic BD compared with nonpsychotic BD, but the findings are inconsistent (Bora, 2018). Patients with psychotic BD also seem to have an increased suicide rate, poorer general health and more comorbid somatic illnesses (Baldessarini & Tondo, 2020; Schaffer et al., 2015). Thus, psychotic symptoms appear to be an important predictor of the clinical outcome of BD. However, the reported prevalence of psychotic symptoms in BD patients varies because of differences in sampling and other methodological issues.

To the best of our knowledge, only one previous systematic review has been published on psychotic symptoms in BD. This review dates from 2007 covering studies intil 2005, with the reported pooled lifetime prevalence of 60% mainly based on inpatient data from patients with mania (Goodwin & Jamison).

Many new studies have been published on BD since then, including information on different subtypes and on different polarities other than mania. Thus, our aim is to systematically review and meta-analyse the lifetime prevalence (‘history of psychosis’) and point prevalence (‘current psychotic symptoms’) in BD, hence differentiating between types of BD (BDI *v.* BDII), mood episodes (manic, depressive or mixed sample), setting (patient samples such as inpatient and outpatient settings, as well as from the general population in community samples) and diagnostic system used (ICD *v.* DSM) for when there are available data.

## Methods

### Protocol and registration

This systematic review was performed according to the Preferred Reporting Items for Systematic Reviews and Meta-analyses (PRISMA) guidelines, see online for Supplementary Material 1a & 1b: PRISMA 2020 Abstract checklist and Checklist. The search protocol was prospectively published in PROSPERO, project number CRD42017052706 (Aminoff, Melle, Ødegaard, & Onyeka, 2017).

### Search strategy

A literature search was conducted with assistance from a senior librarian (MØ) at the Medical Library at the University of Oslo. We searched Medline, Embase, PsycINFO (Ovid interface) and Cochrane Library (Wiley) on 5 August 2021; no date limitation was set. The search was limited to English, Norwegian, Danish and Swedish. Both text words and subject headings were used. The search terms were variations of ‘bipolar disorder’, ‘hypomanic’, ‘manic’ or ‘cyclothymic’; combined with either ‘psychotic’, ‘psychosis’, ‘hearing voices’, ‘auditory hallucinations’, ‘paranoid behaviour’ or ‘delusion’; and ‘adults’. After removing duplicates, a total of 2807 references were included for further screening. The search is published online on Open Science Framwork (OSF) in accordance with PRISMA 2020 recommendations, see (Ødegaard & Aminoff, 2022).

### Inclusion and exclusion criteria

The inclusion criteria for the systematic review and meta-analyses were as follows: (1) published papers containing data on patients with (BDI, BDII or BD not otherwise specified); (2) restricted to adult BD (aged 18–64 years); (3) papers written in English or a Scandinavian language and (4) studies that reported a proportion of current psychotic symptoms or history of psychotic symptoms. Psychotic symptoms were limited to hallucinations or delusions; however, there were no rules for how the presence of psychotic symptoms should be assessed; 5) we included both observational and experimental studies, all considered cross-sectional due to the nature of the data.

The exclusion criteria were as follows: studies with (1) preselected groups or matching to ensure equal proportions of psychotic and nonpsychotic BD; (2) unknown proportions of schizoaffective disorder bipolar type included in the sample; (3) unknown distribution of BDI *v.* BDII patients in sample; (4) too broad or narrow definition of psychotic symptoms (e.g. limited to mood-incongruent delusions or including disorganised symptoms); (5) conference abstracts; (6) papers where the presence of psychotic symptoms were not mentioned in abstract and (7) studies not subject to peer review.

Many studies did not report the age groups included. In such cases, the mean age was identified, and if the patient population based on this seemed most likely to be between 18 and 64 years, the studies were included. If the mean age was higher than 50 years, with large standard deviations of more than 15 years, the studies were excluded.

#### Samples and types of studies

There were no limitations regarding the type of study population; thus, the current study included a broad range of studies from a variety of settings. We also included studies that established the diagnosis in different ways, varying from standard clinical diagnoses with estimations of psychotic symptoms through chart reviews and registers to diagnostic and symptom assessment procedures using structured interviews and rating scales. Evaluation of the quality of data was, however, conducted and taken into consideration in the review process and in the analyses.

### Study selection, data extraction and data analysis

The retrieved records were first screened by title and abstracts, and the full text was subsequently read for relevance using the predefined inclusion criteria, which was done independently by two of the authors (INO and SRA). Any disagreements or discrepancies were resolved through discussions until consensus was reached (six studies). If there was more than one study reporting from the same sample, we excluded the smallest or one with the least amount of descriptive information. Author SRA extracted data from each study using a data extraction form, including information about the country where the study was conducted, study characteristics, participant characteristics, diagnostic system and diagnostic procedure, type of BD, the method for assessment of psychotic symptoms and the proportion of either a history of psychosis or current psychotic symptoms or both.

### Quality assessment

Quality assessment was done using the Newcastle–Ottawa Scale for cross-sectional studies (Wells et al., 2000), which was customised to fit the purpose of the present study. The adaptation of the scale is available online as Supplementary Material 2: Adapted Newcastle Ottawa Scale. The rating was performed by two independent raters, where the first author evaluated all studies and the co-authors IM, INO, TVL, OAA, KLR and CS each assessed a proportion of the studies. Each study was assigned a score between 0 and 7, where studies with a score of 3 or less were considered being a low quality, 4–5 moderate quality and 6–7 high quality. In the case of discrepancies between the raters, the quality ratings were discussed, and a consensus rating was assigned. Studies of low quality were included in the overall meta-analyses, but follow-up analyses were made to investigate to what extent the results changed if the low-quality studies were removed. The quality assessment can be found online in Supplementary Material 3: Quality Assessment of included studies.

### Analysis

Meta-analyses were performed using Comprehensive Meta-Analysis version 3 software (Borenstein, Hedges, Higgins, & Rothstein, 2013). The pooled lifetime and point prevalence of psychotic symptoms were calculated using random effects meta-analysis, here expressed as percentage and 95% confidence interval (CI). Heterogeneity between studies was measured using the *I*^2^ statistic, which describes the proportion of total variance attributed to variance in the true effect (Borenstein, Hedges, Higgins, & Rothstein, 2009).

For estimates of the point and lifetime prevalence, we performed two main meta-analyses on the samples based on DSM and ICD diagnoses separately. There were too few ICD studies for further subgroup analyses. The DSM sample was divided into subgroup analyses, whenever data were available, and this was based on the subtype of BD (BDI, BDII), patient setting (inpatient, outpatient and community setting) and symptom presentation (mania, mixed or depression).

We performed sensitivity analyses in which we removed studies of low quality, with unstructured diagnostic assessment and/or unclear age range in the studies and looked for major changes in the results.

### Assessment of publication bias

Publication bias was assessed for each group of studies by visual inspection of funnel plots with trim and fill adjustment. Whenever a funnel plot showed indices of asymmetry and the Egger test was significant, we performed subgroup analyses (Borenstein et al., 2009).

## Results

### Search results

**Fig. 1.**
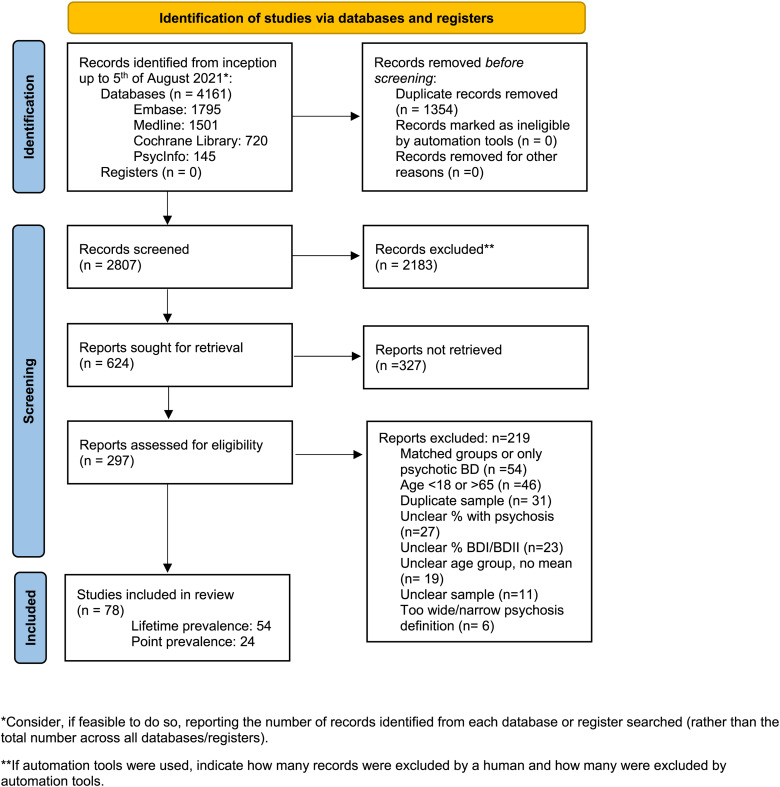
PRISMA 2020 flow diagram for new systematic reviews which included searches of databases and registers only.

Figure 1 shows our searches and selection of relevant papers. From the original 2807 studies, after screening by two independent researchers (SRA and INO), a total of 624 studies were selected for reading the full text, and data were finally extracted from 297 studies after reading the full-text papers. In the end, we included 78 studies.

There were 54 studies (*n* = 23 461) reporting data on the lifetime prevalence of psychotic symptoms in BD, for an overview of included studies see online Supplementary Material 4: Studies included in the meta-analyses of the lifetime prevalence of psychotic symptoms in bipolar disorders (Aedo et al., 2018; Altindag, Yanik, & Nebioglu, 2006; Atagun et al., 2018; Balzafiore et al., 2017; Berkol, Kirli, Islam, Pinarbasi, & Ozyildirim, 2016; Bolhuis et al., 2021; Burton et al., 2018; Cao et al., 2014; Davila et al., 2006; Dell'Osso et al., 2010, 2015; Ekman et al., 2017; Ernst & Goldberg, 2004; Finseth, Morken, Andreassen, Malt, & Vaaler, 2012; Fuentes, Rizo-Mendez, & Jarne-Esparcia, 2016; Gesi et al., 2016; Goldberg et al., 1999; Gonzalez-Pinto et al., 2011; Grande et al., 2017; Grigoroiu-Serbanescu et al., 2010; Jamra et al., 2010; Javadapour et al., 2010; Karakus & Tamam, 2011; Kent et al., 2020; Kirov & Murray, 1999; Knorr et al., 2021; Laidi et al., 2015; Lee & Kim, 2011; Mantere et al., 2004; Miller et al., 2018; Moon et al., 2012; Nardi et al., 2008; Neves, Malloy-Diniz, Barbosa, Brasil, & Correa, 2009; Newport et al., 2012; Ong et al., 2012; Ortiz et al., 2021; Østergaard, Bertelsen, Nielsen, Mors, & Petrides, 2013; Pacchiarotti et al., 2011; Parker et al., 2013, 2020; Patterson, Sandman, Ring, Jin, & Bunney, 2009; Prabhavathy, Kuruvilla, Ravindren, Ganesh, & Midhun, 2017; Sanchez-Morla et al., 2019; Sarrazin et al., 2018; Savitz et al., 2015; Simonsen et al., 2011; Talati et al., 2013; Tost et al., 2010; Trotti et al., 2020; Tundo et al., 2015; Van Der Werf-Eldering et al., 2011; Van Rheenen et al., 2017; Yazici, Kora, Ucok, Tunali, & Turan, 1999; Yilmaz, Yavuz, Altinbas, Lordoglu, & Kurt, 2015).

There were 24 studies reporting data on the point prevalence of psychotic symptoms in BD. For an overview of included studies, see online Supplementay Material 5: Studies included in the meta-analyses of the point prevalence of psychotic symptoms in bipolar disorders (Abulseoud et al., 2020; Asaad et al., 2014; Azorin, Adida, & Belzeaux, 2015; Azorin, Akiskal, & Hantouche, 2006; Basterreche et al., 2008; Benazzi, 2001; Bonnin et al., 2015; Caldieraro et al., 2017; Carroll, Vohs, O'Donnell, Shekhar, & Hetrick, 2007; Ciapparelli et al., 2001; Fiedorowicz et al., 2011; Goldberg & Harrow, 2004; Guven, Kesebir, Demirer, & Bilici, 2015; Kessing, 2004; Kessing, Jensen, & Christensen, 2008; Levy & Weiss, 2010; Lindenmayer, Bossie, Kujawa, Zhu, & Canuso, 2008; Lundin, Bartolomeo, O'Donnell, & Hetrick, 2018; Novis et al., 2014; Reddy, Meyer, Wittlin, Miller, & Weinstock, 2017; Salem et al., 2019; Samalin et al., 2014; Schwartzmann et al., 2007; Yildiz, Guleryuz, Ankerst, Ongur, & Renshaw, 2008).

### Lifetime prevalence of psychotic symptoms in BD

The characteristics of the included studies giving data for lifetime prevalence are shown in the online Supplementary Material 4. Among these 54 studies, 26 were conducted in Europe, 17 in the Americas, 4 in Asia and 3 in Australia, and four of the studies included participants from multiple countries. The studies' quality assessment scores ranged from 2 to 7. A total of 13 (24%) studies were of low quality, 34 (63%) were of moderate quality and 7 (13%) were of high quality (Table 1).

**Table 1.** Results from meta-analyses of the prevalence of psychotic symptoms in bipolar disorders

Analysis	*N*	Number of studies	Percentage	95% CI	*I*^2^
**Lifetime prevalence**					
1.0 Total DSM sample lifetime prevalence	8013	51	57	52–62	94.15
1.1 ≥Moderate quality	6921	39	56	50–61	94.25
1.2 Structured diagnosis	7052	44	55	50–60	93.53
1.3 Defined age span	2432	19	55	47–63	92.83
1.4 Inpatient samples	890	8	68	56–78	90.99
1.5 Outpatient samples	3848	20	57	48–65	95.93
1.6 *Community samples*	NA				
2.0 Total ICD sample lifetime prevalence	15 448	3	23	17–31	92.52
3.0 Total DSM bipolar type I (BDI)	3316	26	67	61–72	87.97
3.1 ≥Moderate quality	2926	20	63	57.5–68	89.27
3.2 Structured diagnosis	2804	21	62	57–67.5	85.45
3.3 Defined age span	745	10	55	45–64	82.89
3.4 Inpatient BDI	684	7	71	61–79	84.31
3.5 Outpatient BDI	481	5	73.5	51–88	93.81
4.0 Total bipolar type II (BDII)	704	7	19	13–29	82.63
4.1 ≥Moderate quality	499	4	22	14–33	82.09
4.2 Structured diagnosis	470	5	23	14–35	81.28
4.3 Defined age span	NA				
4.4 *Inpatient BDII*	NA				
4.5 Inpatient/outpatient BDII	NA				
4.6 Outpatient BDII	494	3	13	8–20	69.55
**Point prevalence**					
5.0 Total DSM sample point prevalence	4856	20	41.5	32–53	97.34
5.1 ≥Moderate quality	3359	12	38	27–50	97.30
5.2 Structured diagnosis	2702	12	37	25–50	96.90
5.3 Defined age span	1263	11	45	32–59	97.09
5.4 Inpatient samples	2947	9	54	39–68	97.93
5.5 Outpatient samples	1178	5	20	11–33	93.82
5.6 *Community samples*	NA				
5.7 Current mania*	2062	6	57	47–66	92.34
5.8 Current depression	780	3	13	7–23	91.02
5.9 *Current mixed*	NA				
6.0 Total ICD sample point prevalence	NA				
7.0 Total DSM bipolar type I (BDI)	3099	14	51	40–61	96.06
7.1 ≥Moderate quality	1602	6	54	41–67	92.98
7.2 Structured diagnosis	1572	8	48	35–61	92.24
7.3 Defined age span	1099	8	51	35–66	96.28
7.4 Inpatient BDI	2669	8	58	43–72	97.64
7.5 *Outpatient BDI*	NA				
7.6 Current mania BDI*	2062	6	57	47–66	92.43
7.7 *Current depression BDI*	NA				
8.0 *Total BDII point prevalence*	NA				

DSM, Diagnostic and Statistical Manual of Mental Disorders; ICD, International Classification of Diseases and Related Health Problems; NA, not applicable.

*Same analysis.

The pooled lifetime prevalence of psychotic symptoms in the 51 included DSM BD studies (*N* = 8013) was 57% (95% CI 52–62) (Aedo et al., 2018; Altindag et al., 2006; Atagun et al., 2018; Balzafiore et al., 2017; Berkol et al., 2016; Burton et al., 2018; Cao et al., 2014; Davila et al., 2006; Dell'Osso et al., 2010, 2015; Ekman et al., 2017; Ernst & Goldberg, 2004; Finseth et al., 2012; Fuentes et al., 2016; Gesi et al., 2016; Goldberg et al., 1999; Gonzalez-Pinto et al., 2011; Grande et al., 2017; Grigoroiu-Serbanescu et al., 2010; Jamra et al., 2010; Javadapour et al., 2010; Karakus & Tamam, 2011; Kent et al., 2020; Kirov & Murray, 1999; Laidi et al., 2015; Lee & Kim, 2011; Mantere et al., 2004; Miller et al., 2018; Moon et al., 2012; Nardi et al., 2008; Neves et al., 2009; Newport et al., 2012; Ong et al., 2012; Ortiz et al., 2021; Pacchiarotti et al., 2011; Parker et al., 2013, 2020; Patterson et al., 2009; Prabhavathy et al., 2017; Sanchez-Morla et al., 2019; Sarrazin et al., 2018; Savitz et al., 2015; Simonsen et al., 2011; Talati et al., 2013; Tost et al., 2010; Trotti et al., 2020; Tundo et al., 2015; Van Der Werf-Eldering et al., 2011; Van Rheenen et al., 2017; Yazici et al., 1999; Yilmaz et al., 2015).

Of these, in 39 studies with at least moderate quality (*n* = 6921), the pooled prevalence was 56% (95% CI 50–61) (Aedo et al., 2018; Altindag et al., 2006; Balzafiore et al., 2017; Berkol et al., 2016; Burton et al., 2018; Cao et al., 2014; Dell'Osso et al., 2010; Dell'Osso et al., 2015; Ekman et al., 2017; Finseth et al., 2012; Fuentes et al., 2016; Gesi et al., 2016; Goldberg et al., 1999; Gonzalez-Pinto et al., 2011; Grande et al., 2017; Grigoroiu-Serbanescu et al., 2010; Jamra et al., 2010; Karakus & Tamam, 2011; Kirov & Murray, 1999; Laidi et al., 2015; Lee & Kim, 2011; Mantere et al., 2004; Miller et al., 2018; Nardi et al., 2008; Neves et al., 2009; Newport et al., 2012; Ong et al., 2012; Ortiz et al., 2021; Pacchiarotti et al., 2011; Patterson et al., 2009; Prabhavathy et al., 2017; Sanchez-Morla et al., 2019; Sarrazin et al., 2018; Simonsen et al., 2011; Talati et al., 2013; Trotti et al., 2020; Tundo et al., 2015; Van Rheenen et al., 2017; Yazici et al., 1999).

In the 44 studies that used structured diagnostic instruments (*n* = 7052), the pooled prevalence was 55% (95% CI 47–63) (Aedo et al., 2018; Altindag et al., 2006; Atagun et al., 2018; Balzafiore et al., 2017; Berkol et al., 2016; Burton et al., 2018; Cao et al., 2014; Dell'Osso et al., 2010, 2015; Ekman et al., 2017; Ernst & Goldberg, 2004; Finseth et al., 2012; Fuentes et al., 2016; Gesi et al., 2016; Gonzalez-Pinto et al., 2011; Grande et al., 2017; Grigoroiu-Serbanescu et al., 2010; Jamra et al., 2010; Javadapour et al., 2010; Karakus & Tamam, 2011; Kent et al., 2020; Laidi et al., 2015; Lee & Kim, 2011; Mantere et al., 2004; Miller et al., 2018; Nardi et al., 2008; Neves et al., 2009; Newport et al., 2012; Ong et al., 2012; Ortiz et al., 2021; Pacchiarotti et al., 2011; Patterson et al., 2009; Prabhavathy et al., 2017; Sanchez-Morla et al., 2019; Sarrazin et al., 2018; Simonsen et al., 2011; Talati et al., 2013; Tost et al., 2010; Trotti et al., 2020; Tundo et al., 2015; Van Der Werf-Eldering et al., 2011; Van Rheenen et al., 2017; Yazici et al., 1999).

In the 19 studies that stated the age span of included patients (*n* = 2432), the pooled prevalence was 56% (95% CI 50–61) (Altindag et al., 2006; Berkol et al., 2016; Davila et al., 2006; Dell'Osso et al., 2010; Finseth et al., 2012; Fuentes et al., 2016; Gesi et al., 2016; Javadapour et al., 2010; Karakus & Tamam, 2011; Laidi et al., 2015; Mantere et al., 2004; Nardi et al., 2008; Newport et al., 2012; Prabhavathy et al., 2017; Sanchez-Morla et al., 2019; Simonsen et al., 2011; Tundo et al., 2015; Van Der Werf-Eldering et al., 2011; Van Rheenen et al., 2017).

Only three studies included samples based on ICD-10 diagnoses (*N* = 15 448). Here, the pooled lifetime prevalence was 23% (95% CI 17–31) (Bolhuis et al., 2021; Knorr et al., 2021; Østergaard et al., 2013).

Details of the results for all meta-analyses of the prevalence of psychotic symptoms in BD are presented in Table 1.

#### Lifetime prevalence of psychotic symptoms in BDI (DSM)

A total of 26 studies (*n* = 3316) reported specific proportions for a history of psychosis in BDI patients, with a pooled lifetime prevalence of psychotic symptoms of 67% (95% CI 61–72) (Altindag et al., 2006; Cao et al., 2014; Davila et al., 2006; Dell'Osso et al., 2010; Gesi et al., 2016; Goldberg et al., 1999; Gonzalez-Pinto et al., 2011; Grigoroiu-Serbanescu et al., 2010; Jamra et al., 2010; Javadapour et al., 2010; Karakus & Tamam, 2011; Kent et al., 2020; Kirov & Murray, 1999; Laidi et al., 2015; Lee & Kim, 2011; Mantere et al., 2004; Nardi et al., 2008; Ong et al., 2012; Pacchiarotti et al., 2011; Parker et al., 2013; Patterson et al., 2009; Prabhavathy et al., 2017; Sarrazin et al., 2018; Tost et al., 2010; Trotti et al., 2020; Yilmaz et al., 2015).

**Fig. 2.**
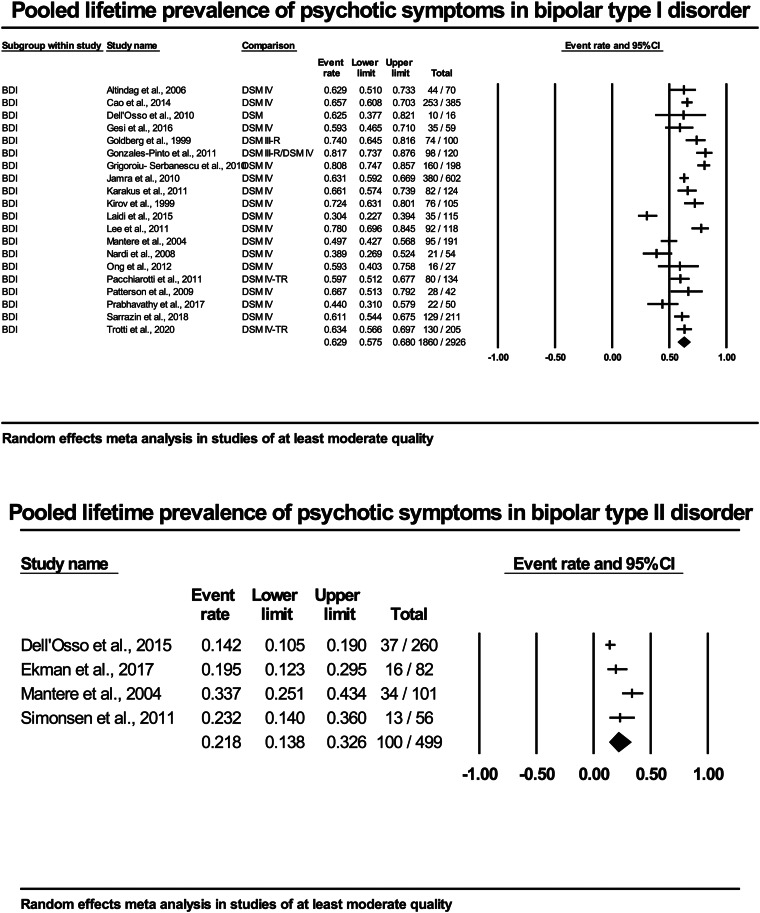
Forest plots of included studies of at least moderate quality in bipolar type I and type II disorder.

For the 20 studies (*n* = 2926) of at least moderate quality, the pooled prevalence was 63% (95% CI 57.5–68) (see Fig. 2).

For the 21 studies (*n* = 2804) using structured diagnostic instruments, the pooled prevalence was 62% (95% CI 57–67.5) (Altindag et al., 2006; Cao et al., 2014; Dell'Osso et al., 2010; Gesi et al., 2016; Gonzalez-Pinto et al., 2011; Grigoroiu-Serbanescu et al., 2010; Jamra et al., 2010; Javadapour et al., 2010; Karakus & Tamam, 2011; Kent et al., 2020; Laidi et al., 2015; Lee & Kim, 2011; Mantere et al., 2004; Nardi et al., 2008; Ong et al., 2012; Pacchiarotti et al., 2011; Patterson et al., 2009; Prabhavathy et al., 2017; Sarrazin et al., 2018; Tost et al., 2010; Trotti et al., 2020).

For the 10 studies (*n* = 745) that stated their included age span, the pooled prevalence was 55% (95% CI 45–64) (Altindag et al., 2006; Davila et al., 2006; Dell'Osso et al., 2010; Gesi et al., 2016; Javadapour et al., 2010; Karakus & Tamam, 2011; Laidi et al., 2015; Mantere et al., 2004; Nardi et al., 2008; Prabhavathy et al., 2017). Forest plots for sub analyses can be found online in Supplementary Material 6.

The pooled lifetime prevalence for the seven studies (*n* = 684) reporting data specifically for inpatient BDI was 71% (95% CI 61–79) (Davila et al., 2006; Goldberg et al., 1999; Grigoroiu-Serbanescu et al., 2010; Lee & Kim, 2011; Pacchiarotti et al., 2011; Prabhavathy et al., 2017; Tost et al., 2010).

The pooled lifetime prevalence for the five studies (*n* = 481) reporting data for BDI treated in outpatient settings was 73.5% (95% CI 51–88) (Dell'Osso et al., 2010; Gonzalez-Pinto et al., 2011; Karakus & Tamam, 2011; Nardi et al., 2008; Yilmaz et al., 2015).

There were no community sample-based studies that met our inclusion criteria.

For more details, see Table 1.

#### Lifetime prevalence of psychotic symptoms in BDII (DSM)

The pooled lifetime prevalence of psychotic symptoms in the seven studies (*n* = 704) with data on BDII was 19% (95% CI 13–29) (Atagun et al., 2018; Dell'Osso et al., 2015; Ekman et al., 2017; Mantere et al., 2004; Moon et al., 2012; Savitz et al., 2015; Simonsen et al., 2011).

When limiting the studies to those four studies of at least moderate quality (*n* = 499), the pooled lifetime prevalence for psychotic symptoms was 22% (95% CI 14–33). For more details, see Fig. 2.

The five studies (*n* = 470) using structured diagnostic instruments had a pooled prevalence of 23% (95% CI 14–35) (Atagun et al., 2018; Dell'Osso et al., 2015; Mantere et al., 2004; Savitz et al., 2015; Simonsen et al., 2011). Forest plots for sub analyses can be found online in Supplementary Material 6.

There were only two studies with clear information about the age span for the included patients (Mantere et al., 2004; Simonsen et al., 2011), which is too few to meta-analyse.

There were no studies specifically of BDII in inpatient settings, while the pooled lifetime prevalence of psychotic symptoms specifically in outpatient BDII samples based on three studies (*n* = 494) was 13% (95% CI 8–20) (Dell'Osso et al., 2015; Ekman et al., 2017; Moon et al., 2012).

Heterogeneity was high throughout most analyses, with lower values for more selected groups, such as BDI and BDII, as expected. For more details, see Table 1.

### Point prevalence of psychotic symptoms in BD

The characteristics of the studies of point prevalence are shown in the online Supplementary Material 5. Among these 24 studies, 12 were conducted in Europe, including Turkey, 10 in the Americas, 1 in Africa and 1 including participants from multiple countries. The studies' quality assessment scores ranged from 2 to 5. Of these, 9 (37.5%) studies had low quality and 15 (62.5%) had moderate quality.

The pooled point prevalence for psychotic symptoms in the 20 studies using DSM BD diagnoses (*n* = 4856) was 41.5% (95% CI 32–52) (Abulseoud et al., 2020; Asaad et al., 2014; Azorin et al., 2006, 2015; Basterreche et al., 2008; Benazzi, 2001; Bonnin et al., 2015; Caldieraro et al., 2017; Carroll et al., 2007; Ciapparelli et al., 2001; Guven et al., 2015; Levy & Weiss, 2010; Lindenmayer et al., 2008; Lundin et al., 2018; Novis et al., 2014; Reddy et al., 2017; Salem et al., 2019; Samalin et al., 2014; Schwartzmann et al., 2007; Yildiz et al., 2008).

Out of these 20 studies, in the 12 studies (*N* = 3359) of at least moderate quality, the pooled point prevalence was 38% (95% CI 27–50) (Abulseoud et al., 2020; Asaad et al., 2014; Azorin et al., 2006, 2015; Basterreche et al., 2008; Benazzi, 2001; Bonnin et al., 2015; Caldieraro et al., 2017; Guven et al., 2015; Lundin et al., 2018; Novis et al., 2014; Samalin et al., 2014).

For the 12 studies (*n* = 2702) using structured assessment, the pooled point prevalence was 37% (95% CI 25–50) (Asaad et al., 2014; Azorin et al., 2006, 2015; Basterreche et al., 2008; Benazzi, 2001; Caldieraro et al., 2017; Carroll et al., 2007; Guven et al., 2015; Levy & Weiss, 2010; Lundin et al., 2018; Schwartzmann et al., 2007; Yildiz et al., 2008).

For the 11 studies (*n* = 1263) that gave information about the age span for the included patients, the pooled point prevalence was 45% (95% CI 32–59) (Abulseoud et al., 2020; Asaad et al., 2014; Azorin et al., 2006; Caldieraro et al., 2017; Guven et al., 2015; Levy & Weiss, 2010; Lundin et al., 2018; Novis et al., 2014; Salem et al., 2019; Schwartzmann et al., 2007; Yildiz et al., 2008).

There were not enough studies to meta analyse the point prevalence for ICD-based studies (Kessing, 2004; Kessing et al., 2008) or studies using research diagnostic criteria (Fiedorowicz et al., 2011; Goldberg & Harrow, 2004). For more details, see Table 1.

#### Point prevalence of psychotic symptoms in BD according to treatment setting (DSM)

A total of nine studies (*n* = 2947) reporting on inpatient samples found a pooled point prevalence of 54% (95% CI 39–68) (Abulseoud et al., 2020; Azorin et al., 2006, 2015; Basterreche et al., 2008; Levy & Weiss, 2010; Lindenmayer et al., 2008; Reddy et al., 2017; Salem et al., 2019; Yildiz et al., 2008).

The five studies (*n* = 1178) based on outpatient samples had a pooled point prevalence of 20% (95% CI 11–33) (Benazzi, 2001; Caldieraro et al., 2017; Novis et al., 2014; Samalin et al., 2014; Schwartzmann et al., 2007).

Three studies (*n* = 780) reported data for current depressive episodes across both BDI/BDII samples, here with a pooled point prevalence of psychotic symptoms of 13% (95% CI 7–23.5) (Azorin et al., 2015; Benazzi, 2001; Caldieraro et al., 2017). There were no studies of current psychotic symptoms in BD community samples.

For more details, see Table 1.

#### Point prevalence of psychotic symptoms in BDI (DSM)

The 14 studies (*n* = 3099) reporting data on BDI samples gave a pooled point prevalence of psychotic symptoms of 51% (95% CI 40–61) (Abulseoud et al., 2020; Azorin et al., 2006; Basterreche et al., 2008; Bonnin et al., 2015; Carroll et al., 2007; Ciapparelli et al., 2001; Guven et al., 2015; Levy & Weiss, 2010; Lindenmayer et al., 2008; Lundin et al., 2018; Reddy et al., 2017; Salem et al., 2019; Schwartzmann et al., 2007; Yildiz et al., 2008).

**Fig. 3.**
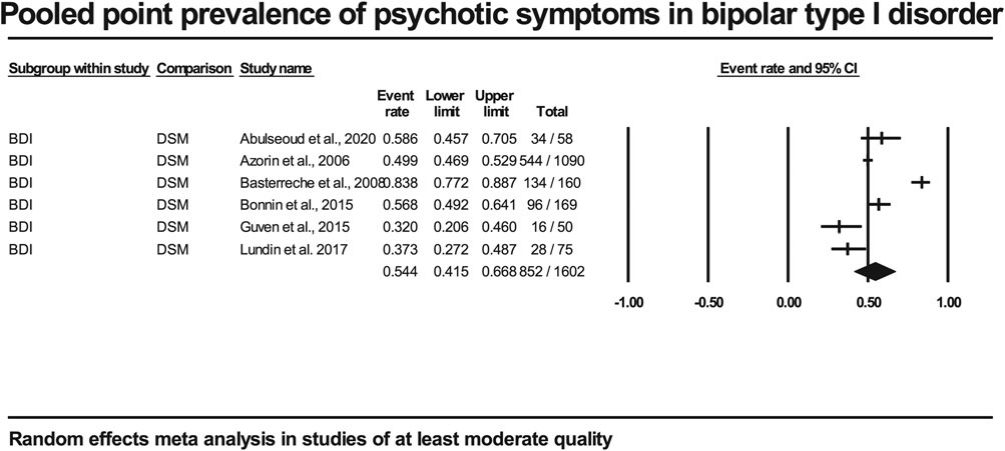
Forest plot of included studies of at least moderate quality in point prevalence of psychotic symptoms in bipolar type I disorder.

In the six BDI studies (*n* = 1602) of at least moderate quality, the pooled point prevalence was 54% (95% CI 41.5–67) (see Fig. 3).

In the eight BDI studies using structured assessment, there was a pooled point prevalence of 48% (95% CI 35–61) (*n* = 1572) (Azorin et al., 2006; Basterreche et al., 2008; Carroll et al., 2007; Guven et al., 2015; Levy & Weiss, 2010; Lundin et al., 2018; Schwartzmann et al., 2007; Yildiz et al., 2008).

The pooled point prevalence in the eight studies (*n* = 2669) of psychotic symptoms in BDI inpatients was 58% (95% CI 43–72) (Abulseoud et al., 2020; Azorin et al., 2006; Basterreche et al., 2008; Levy & Weiss, 2010; Lindenmayer et al., 2008; Reddy et al., 2017; Salem et al., 2019; Yildiz et al., 2008).

There was only one study (*n* = 49, low quality) reporting data for outpatient BDI, here finding a prevalence of 26.5% (Schwartzmann et al., 2007).

The pooled point prevalence for the six studies (*n* = 2062) that reported psychotic symptoms during current manic episodes was 57% (96% CI 47–66) (Abulseoud et al., 2020; Azorin et al., 2015; Basterreche et al., 2008; Bonnin et al., 2015; Guven et al., 2015; Lindenmayer et al., 2008).

No studies reported psychotic symptoms during depressive episodes in BDI. There were not enough studies to estimate the point prevalence of psychotic symptoms in BDI depression or in mixed episodes. For more details, see Table 1.

#### Point prevalence of psychotic symptoms in BDII

We found only one study (*n* = 199, moderate quality) reporting the proportion of current psychotic symptoms in BDII at 8.5% (Benazzi, 2001).

Heterogeneity was high throughout all analyses. For more details, see Table 1.

### Potential publication bias

A funnel plot of the 52 studies assessing the lifetime prevalence of psychotic symptoms in DSM-based BD visually appears symmetrical but with a small difference between the observed and imputed scores, see Supplementary Material 7: Funnel plots of studies included in the estimation of lifetime and point prevalence of psychotic symptoms in bipolar disorders. Egger's regression test for funnel plot asymmetry was significant (*t* = 1.96; df = 50; *p* = 0.05). This suggests that the possibility of publication bias cannot be ruled out. To handle possible publication bias issues, we performed repeated analyses to investigate whether the results changed when the lower-quality studies were removed. The funnel plot of the 39 DSM-based lifetime studies of at least moderate quality visually appears symmetrical, with marginal difference between the observed and imputed scores. The Egger's test for these 39 studies was no longer significant (*t* = 1.28; df = 38; *p* = 0.21) (see Supplementary Material 7). Based on this, we decided to report estimates based on studies of at least moderate quality as our main results.

A funnel plot of the 20 DSM studies assessing the point prevalence of psychotic symptoms in BD visually appears symmetrical, with no difference between the observed and imputed scores (see Supplementary Material 7). Egger's regression test for funnel plot asymmetry was non-significant (*t* = 0.78; df = 18; *p* = 0.44), suggesting a lack of evidence for publication bias.

## Discussion

### Main findings and implications

The present meta-analysis extends the literature about the prevalence of psychotic symptoms in BD by examining both the lifetime and point prevalence of psychotic symptoms across diagnostic subgroups, current clinical status and study settings.

The main finding of the current study is that psychotic symptoms seem to be more common in BD than previously assumed. For BDI, the pooled lifetime prevalence was 63%, and in inpatients alone, it was 71%. The diagnostic systems Diagnostic and Statistical Manual of Mental Disorders (DSM; versions IV and 5) and International Classification of Diseases and Related Health Problems, 10th version (ICD-10) have different criteria regarding the type of psychotic symptoms that are allowed to co-occur with mood episodes in a diagnosis of BD. In ICD-10, any first-rank symptoms during mood episodes would imply a schizophrenia spectrum diagnosis, including (1) delusions of control, influence or passivity, clearly referred to body or limb movements or specific thoughts, actions or sensations; or delusional perceptions; (2) hallucinatory voices giving a running commentary on the patient's behaviour or discussing him/her between themselves or other types of hallucinatory voices coming from some part of the body and/or (3) persistent delusions of other kinds that are culturally inappropriate and completely impossible (e.g. being able to control the weather, or being in communication with aliens from another world) (World Health Organization, 1992). It appears that this specification will not be part of ICD-11 (World Health Organization, 2020) so that first-rank symptoms during mood episodes will no longer exclude a BD diagnosis. In contrast, both DSM-IV and DSM-5 allow for a diagnosis of BD if the first-rank symptoms only occur during mood episodes, here with the exception of hypomania. These exclusion criteria of first-rank symptoms because an ICD-10 BD diagnosis could theoretically lead to a lower prevalence of psychotic symptoms in ICD-10 BD-samples (1992) compared with ICD-11, DSM-IV or DSM-5 (American Psychiatric Association, 2013). The previous estimates were 60% in inpatient BDI (Goodwin & Jamison, 2007).

For the first time, this meta-analysis presents estimates of the pooled lifetime prevalence of psychotic symptoms in BDII. Because the diagnostic criteria do not allow for the presence of psychotic symptoms in hypomanic phases, reports of psychotic symptoms in BDII will necessarily occur in depressive episodes. We were able to identify only seven relatively small studies, indicating a lifetime prevalence of psychotic symptoms in BDII varying between 13% and 29%, depending on the type of treatment setting and study quality (Atagun et al., 2018; Dell'Osso et al., 2015; Ekman et al., 2017; Mantere et al., 2004; Moon et al., 2012; Savitz et al., 2015; Simonsen et al., 2011). This estimate may be somewhat low when considering that one previous systematic review and meta-analysis on the prevalence of psychotic symptoms in major depressive disorder estimated lifetime prevalence rates of 28% in overall patient samples and 42% in inpatient samples (Jääskeläinen et al., 2018); in addition, psychotic symptoms are believed to be more common in bipolar depression than unipolar depression (Souery et al., 2011).

The current study also demonstrates a high point prevalence of psychotic symptoms in BD: more than half (54%) of BD inpatients experience current psychotic symptoms, irrespective of the polarity of the episode. As expected, the point prevalence of psychotic symptoms in outpatients is lower than in inpatients. This is in line with the notion that psychotic symptoms are markers of a more severe episode and lower severity of patients in outpatient than inpatient settings (Azorin et al., 2007). However, there is also a possibility that psychotic symptoms are independent of the severity of the affective symptoms and may by themselves contributing to a more severe episode and the need for hospitalisation (Dubovsky, Ghosh, Serotte, & Cranwell, 2021). In addition, indications that about one in five outpatients with BD experience current psychotic symptoms suggest that clinicians working in outpatient settings should pay attention to possible psychotic symptoms, in addition to mood swings.

Another main finding is limited knowledge about psychotic symptoms in depressive episodes in BD. This was the case in both BDI and BDII and in- and outpatients. This is of clinical importance, given the potentially increased suicide risk in psychotic depression and psychotic mixed episodes (Dell'Osso et al., 2000). More specifically, there seems to be a potential association between, for example, delusions of guilt and suicidality (Fredriksen et al., 2017; Kuperberg et al., 2021), and the potential lack of systematic investigation of such symptoms may be a problem.

We did not meet our aim of reporting the prevalence rates in community samples, either for the lifetime or point prevalence, because of a lack of studies. We found one study, which was excluded from the meta-analysis because of the advanced age of the participants. This study reported a lifetime prevalence of 11% for psychotic symptoms and point prevalence of 4% in previously undiagnosed individuals in the community meeting BD diagnostic criteria (Tuulio-Henriksson et al., 2011).

The ICD-10-based studies are few in number and indicate considerably lower prevalence rates than DSM-based studies. There are several possible reasons for this. First most of the ICD-based studies used the standard discharge diagnosis retrieved from medical records provided by real-world clinical assessments and routine clinical reports, which may be biased by the focus of clinicians on mood disorders. If psychotic symptoms were not systematically addressed, this could lead to psychotic symptoms being under-reported. Another possible reason is that studies using ICD-10 diagnoses do not allow for first-rank psychotic symptoms in BD (including mainly 1) delusions of control, influence or passivity; 2) running commentary hallucinatory voices; and/or 3) other persistent culturally inappropriate or impossible delusions of other kinds (World Health Organization, 1992), yielding diagnoses other than BD in such cases. Because of the lack of high-quality research using standardised diagnostic instruments within the ICD framework, we do not know how common psychotic symptoms are in ICD-10-defined BD.The fact that these first-rank symptoms were allowed as per DSM-IV and 5 (American Psychiatric Association, 2013) could partly explain the discprepancies observed. Future studies based on ICD-11 (World Health Organization, 2020) for which it appears first-rank symptoms no longer exclude BD will provide further clarity.

The clinical implications of the current study indicate that clinicians treating BD should also have knowledge on how to assess and treat psychotic symptoms. Training all clinicians in outpatient services to detect and treat psychotic symptoms is resource demanding, which could be an argument for treating BD patients in specialised BD outpatient units. Indeed, such specialised units appear to reduce the need for hospitalisations, the costs related to treatment and patient satisfaction (Henry et al., 2017; Kessing et al., 2013). Alternatively, BD patients with psychotic symptoms could be treated in units specialised in the treatment of psychotic disorders. Another possibility is to develop shared units for BD and psychotic disorders, in particular in more rural areas.

### Strengths and limitations

#### Strengths

The present systematic review and meta-analysis on both lifetime and point prevalence of psychotic symptoms in different subtypes of BD across types of settings has addressed a gap in the literature.

#### Limitations

There is considerable heterogeneity in our analyses, which calls for caution in interpreting the results. BD is a highly heterogeneous disorder with large variations in symptom presentation, level of functioning and help-seeking behaviour. Because of this natural heterogeneity, it is difficult to disentangle what is a potential cause of publication bias and what is because of the natural heterogeneity of the BD population. Furthermore, most studies have reported on combined samples with BDI and BDII, without reports for diagnostic subgroups. Heterogeneity was handled using random effect models. In general, the quality of the studies reporting point prevalence was lower than for those reporting lifetime prevalence. A considerable percentage (37.5%) of the point prevalence studies were of low quality, which caution interpretability of the results. This was solved by conducting sensitivity analyses, and by reporting results based on moderate quality.

We did not include paediatric or geriatric samples, which could be of interest for further studies. Another weakness is the language restriction, so studies in languages other than English are missing (we did not include any studies in one of the Scandinavian language). We did not find any studies reporting on certain lifetime (community samples, inpatient BDII) and point prevalence subgroups (current depressive episodes in BDI, current mixed episodes, outpatient BDI and BDII in general).

## Conclusion

The results from our systematic review and meta-analysis of the *lifetime prevalence* of psychotic symptoms in BD indicates that in BDI patient populations close to 3/4 had psychotic symptoms during their lifetime. In studies of at least moderate quality, the pooled lifetime prevalence in BDI in general was 63% and 22% in BDII. As for point prevalence, more than half of BD inpatients and BDI patients were found to be experiencing psychotic symptoms in a given episode; this number was roughly one in five in an outpatient setting. Taken together, this suggests that psychotic symptoms could be more common in BD than previously reported. However, there are not enough studies of important settings, such as community samples, and for bipolar depression in general. This lack of studies should be systematically addressed in future studies because psychotic symptoms in BD are a major risk factor for poor outcome and suicidality.

## Supplementary material

For supplementary material accompanying this paper visit https://doi.org/10.1017/S003329172200201X. click here to view supplementary material
