# Supplementary material for: Lifetime and point prevalence of psychotic symptoms in adults with bipolar disorders: a systematic review and meta-analysis
Source: Psychol Med. 2022 Aug 26;52(13):2413–25. doi: 10.1017/S003329172200201X (PMC9647517; doi:10.1017/S003329172200201X)
Supplement: Supplementary file 1 [file S003329172200201Xsup001.zip › S003329172200201Xsup001.pptx]

## Slide 1
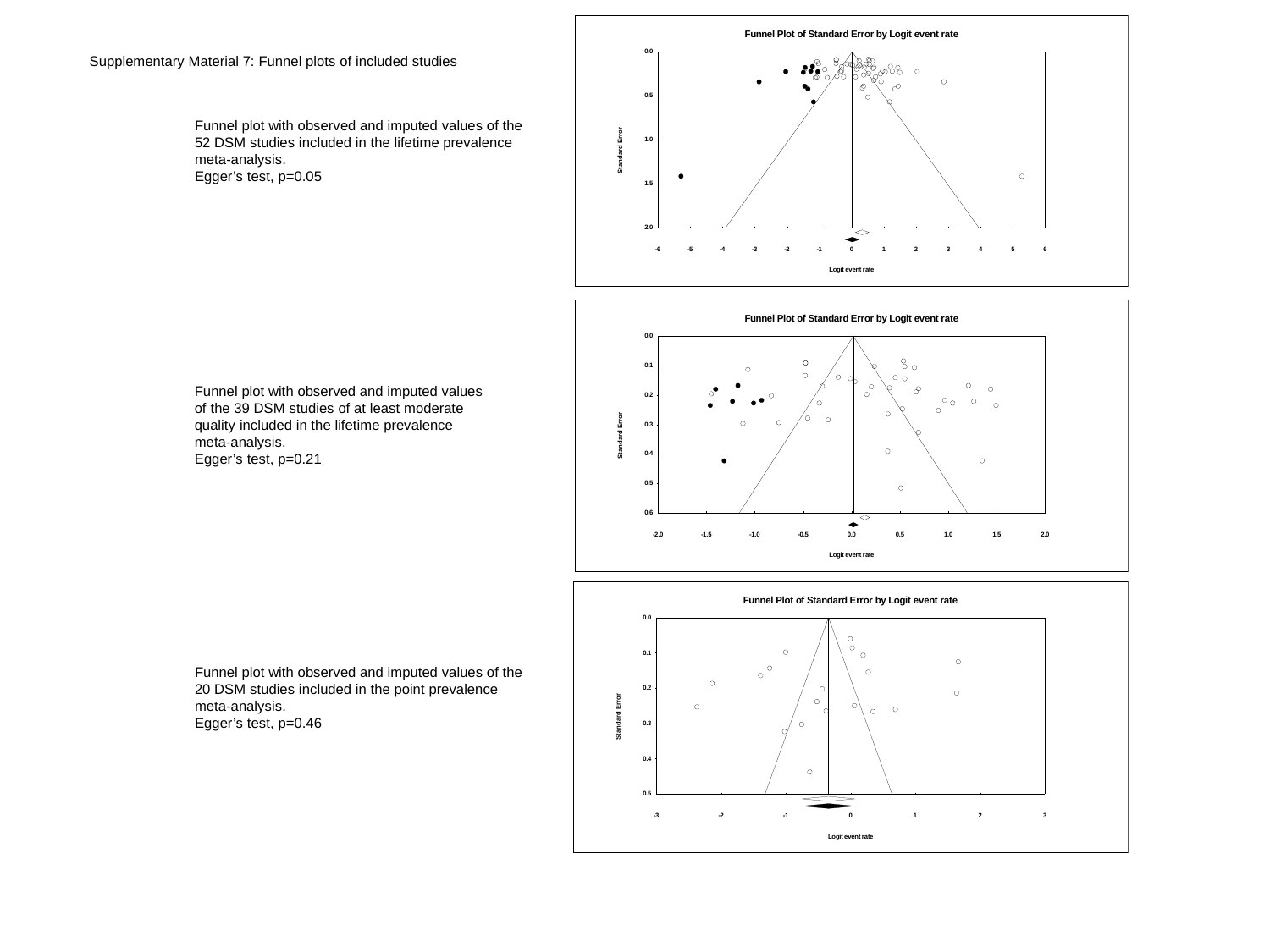

Supplementary Material 7: Funnel plots of included studies
Funnel plot with observed and imputed values of the 52 DSM studies included in the lifetime prevalence meta-analysis.
Egger’s test, p=0.05
Funnel plot with observed and imputed values of the 39 DSM studies of at least moderate quality included in the lifetime prevalence meta-analysis.
Egger’s test, p=0.21
Funnel plot with observed and imputed values of the 20 DSM studies included in the point prevalence meta-analysis.
Egger’s test, p=0.46
