# Supplementary material for: Lifetime and point prevalence of psychotic symptoms in adults with bipolar disorders: a systematic review and meta-analysis
Source: Psychol Med. 2022 Aug 26;52(13):2413–25. doi: 10.1017/S003329172200201X (PMC9647517; doi:10.1017/S003329172200201X)
Supplement: Supplementary file 1 [file S003329172200201Xsup001.zip › S003329172200201Xsup002.docx]

**Supplementary Material 6: Sub analyses in a) lifetime prevalence of bipolar type I disoder; b) lifetime prevalence of bipolar type II disorder; and c) point prevalence of bipolar I disorder**

1. **lifetime prevalence of bipolar type I disoder, sub analyses**

1. **lifetime prevalence of bipolar type II disorder, sub analyses**

1. **point prevalence of bipolar I disorder, sub analyses**
