# Supplementary material for: Lifetime and point prevalence of psychotic symptoms in adults with bipolar disorders: a systematic review and meta-analysis
Source: Psychol Med. 2022 Aug 26;52(13):2413–25. doi: 10.1017/S003329172200201X (PMC9647517; doi:10.1017/S003329172200201X)
Supplement: Supplementary file 1 [file S003329172200201Xsup001.zip › S003329172200201Xsup005.docx]

**Supplementary Material 2: Adapted Newcastle-Ottawa Quality Assessment Scales**

Aminoff et al. (2002), Lifetime and point prevalence of psychotic symptoms in adults with bipolar disorders: a systematic review and meta-analysis.

**Cross-Sectional Studies**

All studies that report findings from one time point are here considered cross-sectional (also case-ctrl and cohort studies that report baseline or outcome data but are assessing something else by nature)

**Selection (maximum 4)**

1. Representativeness of the sample

a. Truly representative of the average in the target population (i.e. catchment area) **

b. Somewhat representative of the average in the target population (consecutive inclusion, multisenter studies) *

c. Selected group of users (for example fertile females)

d. No description of the sampling strategy

2. Sample size

a. Justified and satisfactory *

b. Adequately powered to detect a difference (approximately 100)*

c. Not justified

3. Ascertainment of the exposure (Bipolar disorder)

a. Structured clinical interview (for example SCID/DIGS/MINI plus)*

b. Expert clinician/consensus diagnosis*

c. Medical records, chart reviews, register studies

d. No description

**Outcome (maximum 2)**

5. Assessment of the outcome (psychosis)

a. Clear definition and described method of assessment*

b. Structured interview that provide the information, but not stated explicit in manuscript (for example SCID) *

c. Reported but not how assessed

d. No description

6. Statistical test

a. The statistical test used to analyze the data is clearly described and appropriate (proportion of sample with psychosis and total N reported)*

b. The statistical test is not appropriate, not described or incomplete

**(**Adapted from Wells, G. A., Shea, B., O’Connell, D., Peterson, J., Welch, V., Losos, M., & Tugwell, P. (2000). The Newcastle-Ottawa Scale (NOS) for assessing the quality of nonrandomised studies in meta-analyses)
