# Supplementary material for: Lifetime and point prevalence of psychotic symptoms in adults with bipolar disorders: a systematic review and meta-analysis
Source: Psychol Med. 2022 Aug 26;52(13):2413–25. doi: 10.1017/S003329172200201X (PMC9647517; doi:10.1017/S003329172200201X)
Supplement: Supplementary file 1 [file S003329172200201Xsup001.zip › S003329172200201Xsup006.docx]

**Supplementary Material 3: Quality assessment of included studies**

1. Quality assessment of studies included in meta-analyses of lifetime prevalence of psychotic symptoms of bipolar disorders

1. Quality assessment of studies included in meta-analysis of point prevalence of psychotic symptoms in bipolar disorders.
