# Supplementary material for: Lifetime and point prevalence of psychotic symptoms in adults with bipolar disorders: a systematic review and meta-analysis
Source: Psychol Med. 2022 Aug 26;52(13):2413–25. doi: 10.1017/S003329172200201X (PMC9647517; doi:10.1017/S003329172200201X)
Supplement: Supplementary file 1 [file S003329172200201Xsup001.zip › S003329172200201Xsup007.docx]

**Supplementary Material 4: Studies included in the meta-analyses of the lifetime prevalence of psychotic symptoms in bipolar disorders**
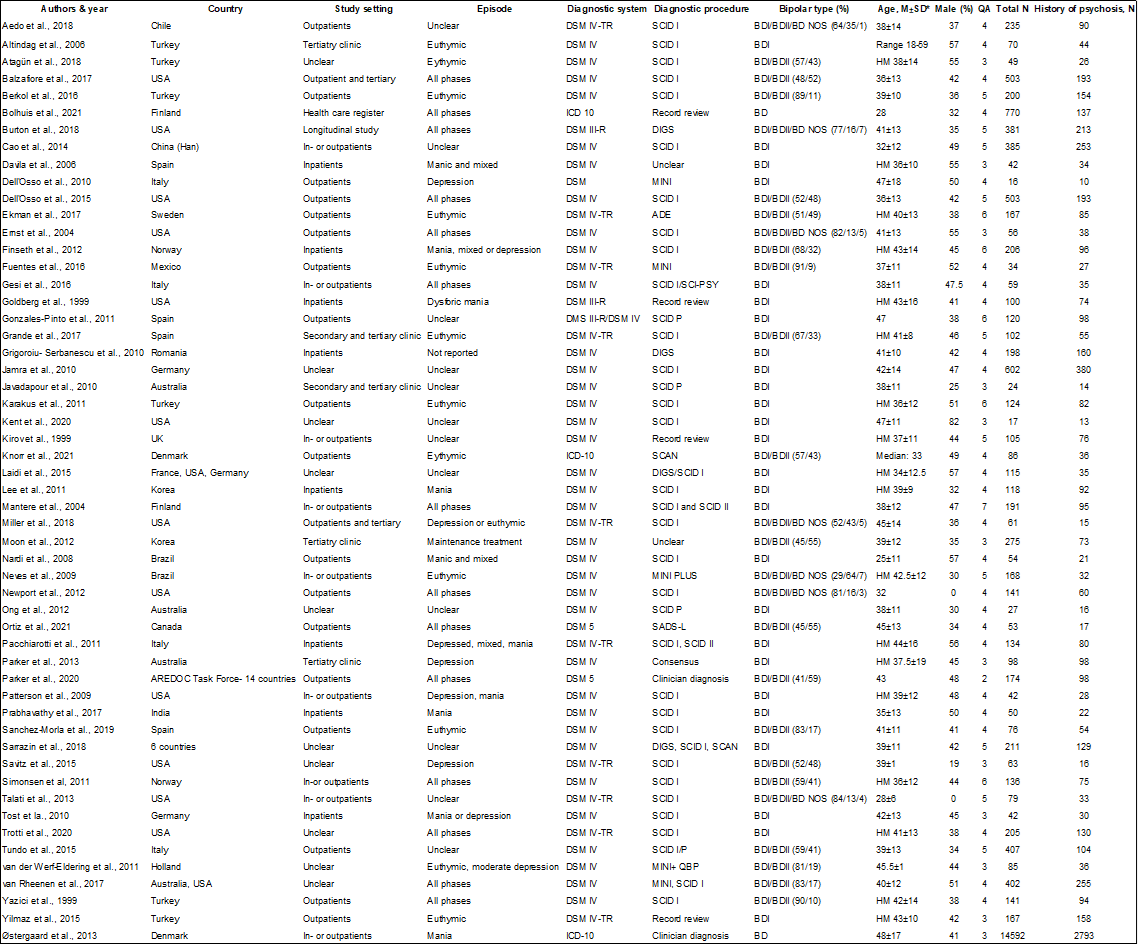


**Abbreviations: ADE**: Affective Disorder Evaluation, **DIGS**: The Diagnostic Interview for Genetic Studies**, DSM**: Diagnostic and Statistical Manual of Mental Disorders, **HM**= Highest mean in a subgroup, when age is not reported for the whole sample**, ICD**: International Statistical Classification of Diseases and Related Health Problems, **MINI (PLUS)**: International Neuropsychiatric Interview (Plus), **SADS-L**: Schedule for Affective Disorders and Schizophrenia – Lifetime version, **SCAN**: Schedules for Clinical Assessment in Neuropsychiatry, **SCID I**: Structural Clinical Interview for DSM-IV-Axis I Disorders (P= Patient edition), **SCID II**: Structural Clinical Interview for DSM-IV-Axis II Disorders, **SCI-PSY**: Structured Clinical Interview for the Psychotic Spectrum, **QA:** Quality assessment, **QBP**: Questionnaire for Bipolar Disorders. * When nothing else is reported
