# Supplementary material for: Lifetime and point prevalence of psychotic symptoms in adults with bipolar disorders: a systematic review and meta-analysis
Source: Psychol Med. 2022 Aug 26;52(13):2413–25. doi: 10.1017/S003329172200201X (PMC9647517; doi:10.1017/S003329172200201X)
Supplement: Supplementary file 1 [file S003329172200201Xsup001.zip › S003329172200201Xsup008.docx]

**Supplementary Material 5: Studies included in the meta-analyses of the point prevalence of psychosis in bipolar disorders**

**Abbreviations:** **DSM**; Diagnostic and Statistical Manual of Mental Disorders, **eMINI-PLUS**= Electronic Mini International Neuropsychiatric Interview Plus**, HM**= Highest mean in a subgroup, when age is not reported for the whole sample, **ICD**: International Statistical Classification of Diseases and Related Health Problems, **Md**: Median, **RDC**; Research Diagnostic Criteria, **SADS**: Schedule for Affective Disorders and Schizophrenia**, SCID I**: Structural Clinical Interview for DSM-IV-Axis I Disorders (P/CV= Patient edition/Clinician Version), **QA**: Quality assessment. * When nothing else is reported
